# Supplementary material for: Content-rich biological network constructed by mining PubMed abstracts
Source: BMC Bioinformatics. 2004 Oct 8;5:147. doi: 10.1186/1471-2105-5-147 (PMC528731; doi:10.1186/1471-2105-5-147)
Supplement: Additional File 2 — The original results of the above study (non-essential files are deleted to keep the file size under the limit set by BMC bioinformatics). [file 1471-2105-5-147-S2.bz2 › chilibotAdditionalFile2/dip05/736ID11983079E18094/html/FDX_HSCA.html]

 


 **FDX** and **HSCA** 
  
Found 8 abstracts in PubMed, retrieved 05.  
 

 What does Google say? 
 PDF only 
| .edu only 

---

**Interactive relationship** (e.g. stimulation, inhibition, etc)

**Neutral relationship**- In addition, we have identified previously unreported interactions between IscS and  **Fdx** , IscS and ORF3, IscA and  **HscA** , and  **HscA**  and  **Fdx** .  Ref: 11983079 J Biochem Tokyo , 2002
- The results presented here indicate crucial roles for IscU, HscB,  **HscA** , and  **Fdx**  as central components of the assembler machinery and also provide evidence for interactions among them.  Ref: 11432781 J Biochem Tokyo , 2001
- The proteins encoded by  **hscA**  and hscB, the chaperone HSP66 and the co chaperone HSP20, have been shown to be required for the synthesis of 2Fe 2S clusters on ferredoxin  [ **FDX** ]  in proteobacteria.  Ref: 11689493 Hum Mol Genet, 2001

**Non-interactive relationship** (e.g. studied together, co-existance, homology, etc.)

- Functional assignment of the ORF2 iscS iscU iscA hscB  **hscA**   **fdx**  ORF3 gene cluster involved in the assembly of Fe S clusters in Escherichia coli.  Ref: 10544286 J Biochem Tokyo , 1999
- By coexpressing these plasmids with reporter Fds, we show the iscS, iscA,  **hscA** , and  **fdx**  genes to be required for the assembly of the Fe S clusters.  Ref: 10544286 J Biochem Tokyo , 1999
- Strains with mutations in the iscU, hscB,  **hscA** , and  **fdx**  genes also exhibited conspicuous phenotypical consequences almost identical to one another.  Ref: 11432781 J Biochem Tokyo , 2001
- Thus, the sequential association and dissociation among the IscS, IscU, IscA, HscB,  **HscA** ,  **Fdx** , and ORF3 proteins may be a critical process in the assembly of Fe S clusters.  Ref: 11983079 J Biochem Tokyo , 2002
- We also examined coexpression using the  **fdx**  gene in the isc gene cluster as a reporter Fd and identified iscS, hscB,  **hscA** , and ORF3 as being involved in the assembly of the 2Fe 2S cluster in this protein.  Ref: 10544286 J Biochem Tokyo , 1999
